# Wave frequency selection method for hyperspectral hyperspectral remote sensing image based on SSGIE-KFCM algorithm

**DOI:** 10.1371/journal.pone.0343986

**Published:** 2026-04-17

**Authors:** Dandan He, Chaokui Ning, Hong Li

**Affiliations:** School of Information Engineering, Pingdingshan University, Pingdingshan, China; Hanoi University of Mining and Geology, VIET NAM

## Abstract

In hyperspectral remote sensing image band selection, there exist issues such as poor nonlinear separability, high redundancy, and the tendency of traditional optimization algorithms to get trapped in local optima. In an effort to tackle these obstacles, the research puts forward an improved band selection method based on the concept of Kernel Fuzzy C-Means Clustering Based on Adaptive Step Firefly Algorithm and Information Entropy Guidance (SSGIE-KFCM). The study achieves efficient band screening through a two-stage optimization framework, utilizing a Gaussian kernel function to enable high-dimensional mapping of band feature spaces and employing cross-sampling and information entropy-based grouping strategies for band feature extraction. Considering computational efficiency, an improved firefly algorithm (FA) is introduced to enhance the global optimization search performance of kernel fuzzy C-means clustering. Adjusting the step size of the FA effectively ensures its rapid convergence and the validity of individual position updates. The outcomes indicate that the proposed approach achieves an average band classification accuracy exceeding 90% on both the Indian Pines and Pavia University datasets, with an area under the curve value of 0.958, and consumes only 40% of the time required by traditional methods. Moreover, the improved algorithm proposed in the study exhibits superior discrimination performance across different ground feature bands, with spectral feature computation times of 0.058s and 0.172s, outperforming other comparative algorithms. The proposed method offers a lightweight solution for real-time processing of remote sensing hyperspectral remote sensing image and holds significant engineering value in agricultural monitoring and urban ground feature classification.

## 1. Introduction

With the rapid progress of hyperspectral remote sensing (RS) technology, hyperspectral RS image play an important role in environmental monitoring and disaster warning, and other fields [1]. Hyperspectral RS technology achieves the precise observation capability of “integrating spectra” by obtaining reflection or radiation information of land objects in dozens to hundreds of continuous narrow bands, providing rich spectral features for land object identification and classification [2]. However, while this high-dimensional data characteristic brings information advantages, it also presents significant data processing challenges: On the one hand, hyperspectral images typically contain a significant quantity of highly correlated adjacent bands, leading to a significant increase in data redundancy. On the other hand, the rapid expansion of the number of bands has caused a “curse of dimensionality” phenomenon, which not only greatly increases computational complexity, but may also reduce the accuracy of classification and recognition [3]. However, hyperspectral data has the characteristics of a significant quantity of bands, high redundancy, and overlapping information. How to efficiently select the most representative subset of bands has become a key issue in improving the efficiency and accuracy of RS image processing. This technological challenge is mainly reflected in three dimensions: Firstly, the selected subset of wave segments should preserve the classification and discrimination information of the original data to the greatest extent possible. Secondly, it is necessary to effectively eliminate redundant correlations between bands. Finally, specific requirements for different application scenarios need to be considered, such as atmospheric window transmittance, sensor response characteristics, and other physical constraints. Traditional band selection methods often rely on manual experience or simple statistical features, which are difficult to adapt to high-dimensional data processing requirements in complex scenarios [4]. Some band selection methods tend to choose bands with the highest information content, such as those with high peak values or high variance. However, this strategy may overlook some bands with low information entropy, seemingly smooth but unique recognition ability for specific land cover categories, resulting in information redundancy and suboptimal classification results. For example, in hydrological research, Goodarzi et al. found that snowmelt may be the dominant factor driving flood events in high-altitude areas. Ignoring this decisive factor under specific conditions can seriously affect the accuracy of remote sensing assessments [5]. “Communication image” usually refers to the optical image used to transmit information, and its design is core to encoding and decoding, focusing on the reliability of information transmission rather than spectral authenticity. Classical hyperspectral analysis aims to accurately identify substance components through continuous, fine spectral information. The fundamental goals of the two are fundamentally different from the evaluation system. The essence of the “remote sensing communication image” is a communication carrier, and the spectrum may be greatly compressed or distorted to ensure transmission efficiency. Ordinary hyperspectral data is a scientific observation carrier, pursuing spectral fidelity and integrity for quantitative analysis. The former is a communication service, and spectral information is the price; the latter is a ground object detection service, and spectral information is the core. While providing rich feature information, hyperspectral remote sensing technology also brings “dimensional disasters” and data redundancy problems. The research aims to develop an efficient band selection method, which aims not only to screen out the most informative subset of bands for ground analysis tasks, but also to provide front-end support for efficient data communication. By intelligently reducing the dimensionality of the original data through in-orbit or near-ground platforms, key bands are selected for priority compression and transmission, which can transmit core information as quickly as possible at the lowest bandwidth cost. Therefore, developing an intelligent and adaptive band selection method has important theoretical and practical value. Traditional clustering methods rely on Euclidean distance, which makes it difficult to handle the nonlinear structure of hyperspectral data. Additionally, they are sensitive to initial clustering centers and prone to falling into local optima [6,7]. Heuristic optimization algorithms, such as genetic algorithms and particle swarm optimization, have high computational complexity, slow convergence speed, and are difficult to adapt to large-scale hyperspectral data [8].

In response to the shortcomings of existing band selection methods in regard to computational efficiency and classification accuracy, this study proposes an improvement to the fuzzy c-means clustering algorithm using kernel function (KFCM), and integrates it with the firefly algorithm (FA) to obtain an improved intelligent band selection method (SSGIE-KFCM) for adaptive optimization of hyperspectral data, and verifies its effectiveness in hyperspectral RS image. The study focuses on hyperspectral hyperspectral RS image, with a focus on band selection. The aim is to select the most informative subset from hundreds of bands to improve the efficiency of subsequent classification, recognition, or transmission. The innovation of the research lies in three aspects. Firstly, the introduction of Gaussian kernel function maps band vectors to high-dimensional space, enhancing the nonlinear separability of KFCM. Secondly, the strategy of “cross sampling+information entropy grouping” combines spatial statistics with information theory to construct an initialization framework under the dual constraints of “space information”. Traditional methods either ignore spatial structure or rely solely on information content, while this combination strategy can ensure that cluster centers are widely distributed in the high-dimensional feature space (covering the entire information spectrum from low entropy to high entropy) and originate from spatially representative samples during the initialization stage, providing a more structurally optimal and globally optimal starting point for subsequent kernel clustering. This is a problem driven technological innovation, whose core contribution lies in significantly improving the convergence robustness and efficiency of nonlinear clustering algorithms in hyperspectral band selection tasks. Thirdly, by improving the FA to avoid getting stuck in local optima, the search efficiency and computational performance have been enhanced. The study combines dynamic step size FA with KFCM to solve the nonlinear optimization problem of hyperspectral band selection, providing an efficient and adaptive solution for hyperspectral band selection and technical support for RS intelligent processing and communication optimization.

## 2. Literature review

Hyperspectral RS band selection mitigates the high correlation resulting from spectral continuity through the selection of feature combinations. However, traditional methods that rely on fixed strategies face challenges in comprehensively exploring the entire solution space and dynamically adapting the optimization process. Consequently, the solutions they yield are often local optima. Based on this, Y. Wan et al. proposed using improved particle swarm optimization algorithm and fitness function modeling to achieve adaptive selection of hyperspectral image RS bands, and improving algorithm search performance by dynamically adjusting motion parameters. The findings demonstrated that this method could effectively balance global and local capabilities, and exhibited good band selection effectiveness on RS image datasets [9]. While this method effectively balances global and local search, its performance remains sensitive to the initial particle distribution and can suffer from premature convergence in complex, multi-modal search spaces. Traditional hyperspectral image classification methods often overlook the correlation between local spatial features. To address the redundancy of spectral bands in hyperspectral images, M. Rogers et al. reviewed and compared wavelength selection strategies with spatial features, and concluded that optimizing spatial extraction and multi-band filtering synchronously could improve prediction robustness [10]. X. Li et al. focused on the bottleneck of small sample hyperspectral image classification and summarized that the accuracy ceiling caused by limited training sets could be overcome through the synergy of algorithm amplification and dimensionality reduction [11]. Traditional RS image feature space fusion classification (FSFC) methods suffered from issues such as low classification accuracy and poor application performance. Based on this, Q. Sun et al. proposed an RS image FSFC method utilizing the ant colony optimization (ACO) algorithm. Grounded in the state transition rules of the ACO algorithm, the global optimal path was updated. Additionally, a feature extractor was employed to capture spatial structure, edge, and texture features from RS images, enabling FSFC. The results indicated that this method improved the average classification accuracy (AA) by 9.75% and enhanced the classification speed by 15.6%, effectively boosting the image recognition rate [12]. However, ACO-based methods are known for their high computational cost and slow convergence, especially as the number of bands (i.e., the solution space) grows.

The accuracy of classification is of great significance in the change detection and analysis of multispectral images. M. H. Vahitha Rahman et al. addressed the problem of multispectral RS data processing by combining an adaptive homomorphic filter model and a constrained proximal fuzzy clustering segment approach. The results indicated that this method had generalization ability and was sensitive to initialization and pixel level features, with good clustering performance [13]. S. L. Krishna et al. proposed a fuzzy twin proximal improved kernel neural network model based on deep learning (DL) for hyperspectral image classification. This method demonstrated good classification accuracy on the dataset [14]. Z. Liu Scholar proposed using integrated empirical model decomposition to achieve hyperspectral image decomposition, and processing it using K-means and hierarchical clustering. The outcomes showed that the unsupervised clustering method improved classification accuracy (over 0.90) and effectively addressed the limitations of high noise signal pollution in spectral data [15]. But it still relies on linear distance measurement (Euclidean distance in K-means), which is not sufficient to capture the nonlinear relationships between spectral bands. The high capacity of hyperspectral images makes dataset processing difficult. H. Tulapurkar et al. proposed an attention technique based on multi-head transformers for remote spectral dependency capture, and used wavelet convolutional neural networks for feature extraction. The findings indicated that the proposed algorithm performed well in regard to information conservation and class separation [16]. While achieving high accuracy, these deep learning models typically demand large annotated datasets for training, possess high computational complexity, and their “black box” nature can make the band selection process difficult to interpret.

J. Zhuang et al. proposed a band selection method that integrates multiple features and affine propagation clustering to address the classification and recognition challenges caused by high redundancy and strong correlation in hyperspectral images. This method utilizes gray level co-occurrence matrix to extract texture features and combines Euclidean distance to construct similarity matrix, and then clusters and reduces the dimensionality of bands through affine propagation algorithm. The results show that this method can effectively handle spatial and spectral redundancy, and significantly improve classification accuracy [17]. These methods, while powerful, often treat spatial and spectral feature extraction as separate or sequential steps, potentially missing the complex interplay between them. Moreover, methods like affine propagation can be computationally demanding and sensitive to the “preference” parameter. Considering the problems of high memory consumption and easily disturbed inherent data structure in existing band selection methods, K. Henneberger et al. proposed a band selection model based on tensor low rank and generalized three-dimensional total variation. This method utilizes tensor CUR decomposition to capture low rank structures and improve computational efficiency. The experimental results show that this method exhibits good robustness and accuracy in different noise scenarios [18]. But its complexity might limit its application in scenarios requiring rapid processing. Faced with the challenge of effectively reducing the number of selected spectral bands in a large combination space when evolutionary algorithms handle hyperspectral band selection, M. Wang et al. proposed a three evolution differential evolution mechanism with information interaction. This method groups bands and co evolves them to obtain the optimal subset of bands that meet the conditions for information exchange. The results show that this method outperforms other comparative methods in multiple indicators, significantly reducing the number of selected bands while improving classification accuracy [19]. This grouping strategy, while innovative, relies on random partitioning, which may not effectively capture the intrinsic spectral structure, and the complexity of co-evolution can be a computational bottleneck.

Analysis of previous literature reveals that existing clustering and kernel based methods face significant challenges in accuracy, efficiency, and robustness in hyperspectral band selection: 1. Insufficient handling of nonlinear redundancy: Traditional clustering algorithms such as K-means or fuzzy C-means (FCM) [15] are essentially based on Euclidean distance. This linear indicator cannot effectively simulate the inherent complex nonlinear correlations and redundancies in high-dimensional spectral data, resulting in suboptimal band groups. 2. Extremely sensitive to initialization: Clustering algorithms and their enhanced swarm intelligence optimizers [9,12] are highly sensitive to initial conditions. Improper and random initialization of clustering centers or groups often forces algorithms to enter local optima, seriously affecting the quality of the final selected subset. Current research lacks robust and information aware initialization strategies. 3. Decoupling of kernel methods and clustering: Although kernel methods are known for their ability to map data to a linearly separable feature space, they are typically used as independent preprocessing steps before clustering (such as KPCA). This multi-stage approach leads to decoupling, where the powerful distance metric in the kernel space no longer directly guides the band clustering process itself. This separation leads to information loss and prevents the full potential of the kernel from being realized. 4. Slow and unstable convergence: Introducing swarm intelligence algorithms for optimizing clustering often brings a series of problems, such as slow convergence speed and the need to adjust complex control parameter arrays. This makes the model more complex and may offset the efficiency advantage of clustering based methods.

Therefore, the core challenge lies in designing an integrated framework that can efficiently handle nonlinear redundancy simultaneously, ensure fast and stable convergence, and overcome initialization sensitivity. To fill this gap, the research proposes the SSGIE-KFCM model. This model internally unifies kernel techniques within the fuzzy C-means framework and directly utilizes kernel spatial distance to guide the clustering of nonlinear bands. Meanwhile, to solve the problem of sensitivity to initial values and slow convergence in clustering algorithms, the research designs an entropy guided intelligent grouping strategy to provide high-quality initial solutions, and introduces an adaptive improved firefly algorithm to accelerate and stabilize the optimization process. Aiming at the core pain points of existing technologies in nonlinear processing, convergence efficiency, and initial condition dependence, a band feature selection method has been designed to better handle the nonlinear structure of hyperspectral data and improve the global search ability of the algorithm.

## 3. Design of wave frequency selection algorithm for hyperspectral RS image

### 3.1. Design of wave frequency selection method based on SSGIE-KFCM algorithm

Hyperspectral RS images achieve precise characterization of ground spectral features through hundreds of consecutive narrow bands, with high spectral correlation between adjacent bands and significant information overlap. Moreover, the number of bands is highly correlated with classification accuracy, leading to problems such as large application errors, low band redundancy, and difficulty in meeting real-time requirements in traditional clustering based selection methods [20]. Therefore, the research is based on the FCM algorithm, which allows data points (DPs) to belong to multiple clusters in the form of membership degrees. The fuzzy membership advantage of this algorithm can effectively quantify the correlation strength between pixels and categories, and its unsupervised characteristics can adapt to the high cost of hyperspectral data annotation. Equation (1) represents the objective function expression under FCM minimization [21].


$$J\left(U,V\right)=\sum_{i=1}^{N}\sum_{j=1}^{C}\overset{m}{\underset{ij}{u}}\cdot \overset{\text{2}}{\underset{ij}{d}}$$ (1)

In Equation (1), N is the number of DPs, C is the number of clustering clusters, U is the membership matrix, $u_{ij}$ represents the membership degree of the i th DP to the j th cluster, m is the fuzzy factor, and $d_{ij}$ is the distance from the DP to the cluster center. The sum of all cluster membership degrees for each DP in the membership matrix must be 1. The Lagrange multiplier method can be employed to solve and partially derive Equation (1), and the calculation formula for membership degree can be derived based on it, as shown in Equation (2).


$$u_{ij}=\frac{\text{1}}{\overset{C}{\underset{k=1}{\sum }}\text{(}\frac{d_{ij}}{d_{ik}}{\text{)}}^{\frac{\text{2}}{m-1}}}$$ (2)

In Equation (2), k is the summation index. In the FCM process, after knowing the coordinates of the cluster centers, iterative processing can be achieved by calculating the membership matrix and cluster center matrix until the convergence conditions are met before stopping the calculation. The FCM algorithm can achieve good accuracy in band classification, but to further improve the efficiency of band vector calculation, research has been conducted on improving the FCM algorithm through kernel clustering calculation, that is, using Gaussian kernel functions to achieve feature mapping of band vectors, resulting in the KFCM algorithm [22]. Traditional FCM directly calculates the Euclidean distance of band vectors, which has limited effectiveness for nonlinear separable data. The kernel function can project data onto a high-dimensional feature space through nonlinear mapping, enhancing linear separability [23]. By replacing Euclidean distance with kernel distance, the clustering centers in the original FCM can be removed to obtain an improved objective function, as shown in Equation (3).


$$JK\left(U,V\right)=2\sum_{i=1}^{N}\sum_{j=1}^{C}\overset{m}{\underset{ij}{u}}\left(1-K\left(x_{i},{\tilde{v}}_{j}\right)\right)$$ (3)

In Equation (3), $K\left(x_{i},{\tilde{v}}_{j}\right)$ is the Gaussian kernel function and ${\tilde{v}}_{j}$ is the implicit center. The objective function of KFCM only relies on the kernel matrix, and its membership degree only needs to be calculated for the clustering center in the initialization part. Subsequent iterations can simplify this step. Hyperspectral RS images face a wide range of land types and present a rich variety of spectral feature pixels. However, the randomness of the band feature sampling process makes it ignore the spatial distribution of pixels. Therefore, the study proposes to use cross spatial sampling, and the sampling band $\hat{H}$ can be expressed as Equation (4) [24].


$$\hat{H}=SamPling\text{(H)}$$ (4)

In Equation (4), SamPling represents the sampling process. The IE of hyperspectral image bands is directly proportional to the amount of detail information they contain, and its mathematical expression can be expressed as Equation (5).


$$H\left(x\right)=-\sum_{\omega \in \Omega }p\left(\omega \right)logp\left(\omega \right)$$ (5)

In Equation (5), Ω represents the pixel value space after grayscale conversion, and p(ω) represents the relative likelihood of grayscale ω pixel value distribution. Although this indicator can to some extent characterize the detailed features of spectral bands, directly selecting bands with high entropy rankings as the initial clustering centers can easily cause selection bias, which contradicts the specificity of spectral band clustering regions in hyperspectral images. Specifically, high entropy bands are often concentrated in the local peak areas of the spectral curve, and the continuity of the IE sorting selection method may lead to clustering centers biased towards high information regions, which cannot effectively cover the entire spectral feature space [25]. Therefore, the study proposes to evenly divide the band IE into several groups, extract the band index with the highest IE within each group, and use its corresponding spectral vector as the clustering center. After comprehensive consideration of bands that cannot be evenly grouped, they can be appropriately discarded. Equation (6) can be expressed as the selected band index In.


$$In=max\text{\textbackslash{}hspace\{0.33em}\}inde\overset{m}{\underset{z=1}{x}}\left(GIEz\right)$$ (6)

**Fig 1:**
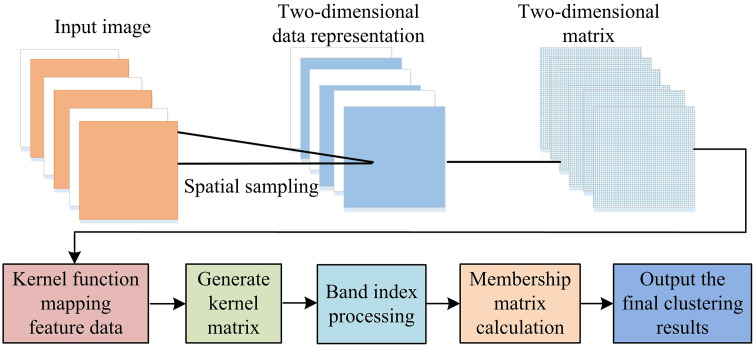
Schematic diagram of SSGIE-KFCM algorithm flow.

In Equation (6), GIEz is the set of group IE, z is the set, and index is the index. Based on the above content, the SSGIE-KFCM algorithm can be obtained. In the SSGIE-KFCM algorithm, feature mapping can be achieved through kernel functions, and band feature extraction can also be achieved through cross sampling and IE calculation. Cross sampling aims to obtain spatially representative pixel samples for calculating information entropy. This method forms a “cross shaped” sampling point set by horizontally and vertically grid sampling the original hyperspectral image with a fixed step size (e.g., 1 sample every 5 pixels), and sets the sampling rate to 40% (for the central row and column) to preserve the spatial structure information of the terrain while reducing the number of pixels required for information entropy calculation. In KFCM, the quality of the initial cluster centers has a decisive impact on the convergence speed and final clustering effect of the algorithm. Traditional random initialization methods cannot guarantee that the initial center can cover the diversity of spectral features in high-dimensional and highly correlated hyperspectral data, which can easily lead to algorithms falling into local optima or slow convergence. The study uses information entropy to measure the amount of information contained in each band, and selects representative and diverse initial bands as cluster centers through a deterministic grouping sampling method. In the image, bands with higher entropy values usually contain richer ground details and texture information. Firstly, spatial cross sampling is performed on the hyperspectral image to reduce computational complexity while preserving spatial structural information. Then, based on the grayscale histogram of sampled pixels, the information entropy of each band is calculated to obtain the entropy value sequence corresponding to the band. Next, the study performs information entropy sorting and grouping. When selecting k bands, all bands are sorted from low to high based on their information entropy values. The sorted band list is evenly divided into k consecutive, non-overlapping groups, ensuring that the bands within each group have similar levels of information content. At the same time, there are significant differences in information content between different groups, covering the entire range from low to high information content. The representative bands of each group are selected as the initial center. After completing the grouping, the study selects a representative band from each group. Afterwards, to ensure that the selected initial centers have the highest information value, the band with the highest entropy value within each group is selected as the representative of that group. The spectral vectors of the selected representative bands constitute the k initial clustering centers of the KFCM algorithm. Fig 1 shows the flowchart of the SSGIE-KFCM algorithm.

In Fig 1, the input image is transformed into a two-dimensional data representation through spatial sampling, and the sampled data is further transformed into a two-dimensional matrix. Afterwards, the data is mapped to a high-dimensional feature space using kernel functions to generate a kernel matrix. The kernel matrix will perform indexing and membership matrix processing, and the band index will select and provide sufficient data to rearrange it into a new matrix. Finally, the clustering results are obtained through the membership matrix and kernel matrix.

### 3.2. Improved band classification design of SSGIE-KFCM based on FA

The SSGIE-KFCM algorithm shows good spectral image processing performance, but it should be noted that the FCM clustering results are significantly affected by the initial results of the membership matrix, and there may be local optimal solution solving situations [26]. Therefore, the study utilized the FA to improve the FCM algorithm, to avoid it falling into local optima and obtain an improved SSGIE-KFCM algorithm. The FA performs global optimization search by simulating the information transmission and attraction mechanism between individual fireflies. In the algorithm, each firefly represents a potential solution, and its luminous intensity is proportional to its fitness value. Fireflies will move towards brighter individuals based on the difference in luminous intensity between themselves and surrounding individuals, thereby exploring and optimizing their search space [27]. The FA searches by simulating the behavior of fireflies attracting companions through luminescence, and their attraction and movement mainly rely on brightness and attractiveness. Equation (7) is the mathematical expression of brightness I and attractiveness β [28].


$$\left\{ \begin{array}{c} @l@I_{a}=f(x_{a}{)}^{-1}\\ \beta \left(r_{ab}\right)={\beta_{0e}}^{-\gamma \overset{\text{2}}{\underset{ab}{r}}} \end{array}\right.$$ (7)

In Equation (7), a and b are the fireflies, $f\left(x_{a}\right)$ is the objective function value of the firefly, $\beta_{\text{0}}$ is the initial attractiveness, γ is the light absorption coefficient, and $r_{ab}$ is the Euclidean distance of the firefly. Individual movement is determined by brightness differences, breaking through the fixed inertia weight limitation of traditional particle swarm optimization algorithms [29]. When firefly a moves towards the brighter one b, the updated position formula can be obtained, as presented in Equation (8).


$$\overset{t+1}{\underset{a}{X}}=\overset{t}{\underset{a}{X}}+\beta \left(r_{ab}\right)(\overset{t}{\underset{b}{X}}-\overset{t}{\underset{a}{X}})+\alpha \overset{t}{\underset{a}{\tau }}$$ (8)

In Equation (8), α is the step size factor, τ is the random perturbation vector, t is the iteration count, $\overset{t}{\underset{a}{X}}$ and $\overset{t}{\underset{b}{X}}$ represents the position of the fireflies a and b during the t th iteration. $\left(\overset{t}{\underset{b}{X}}-\overset{t}{\underset{a}{X}}\right)$ is the direction vector of b → a movement. FA can optimize cluster center initialization to avoid local optima caused by FCM random initialization. It can also enhance clustering robustness by combining firefly brightness information and dynamically adjust membership degrees. FA implements the algorithm by initializing the population, calculating individual fitness values, updating position movements, and outputting results. It assumes that the attraction properties of fireflies are gender independent, and their brightness is determined by the objective function. The traditional FA may have problems such as inappropriate parameter selection and difficulty in solving multiple constraint conditions, and the movement of fireflies is not only affected by the individual with the highest fitness at present [30,31]. The random walk term in the standard FA uses a fixed step factor, which makes it difficult to converge when the algorithm approaches the optimal solution due to the oscillation of the solution near the optimal value caused by a larger step factor; When the algorithm falls into a local optimum, a smaller step size factor cannot provide enough disturbance to escape the trap, weakening the global exploration ability. Therefore, the study replaces the fixed step size factor with an adaptive step size factor that dynamically changes based on the individual strengths and weaknesses of fireflies. For fireflies with high adaptability, it is believed that they are approaching an optimal area, so a smaller step size is given to encourage them to conduct fine searches near their current location. For fireflies with low fitness, it is believed that they may be in a poor area or local optimum, so they are given a larger step size to encourage them to perform large-scale random walks in order to jump out of the current area and explore new possibilities. The adaptive step size mechanism can dynamically balance the global exploration and local mining capabilities of the algorithm, thereby improving convergence speed and solution quality. The study introduces a linear decreasing function based on individual ranking to calculate the adaptive step size factor κ for each firefly. At the beginning of each iteration, all fireflies are sorted in ascending order according to their objective function value f(Xi). $\overset{t}{\underset{a}{\text{rank}}}$ represents the ranking of fireflies. Its mathematical expression is shown in Equation (9).


$$\kappa =\;\alpha_{\text{max}}-\left(\overset{t}{\underset{a}{\text{(rank}}}-1)/(N_{p}-1)\;\right)*(\alpha_{\text{max}}-\alpha_{\text{min}})$$ (9)

In Equation (9), $\alpha_{\text{max}}$ and $\alpha_{\text{min}}$ are the minimum and maximum boundary values of the adaptive step size factor, and $N_{p}$ is the population size of fireflies. The study uses the number of variables in the fitness function as the dimension of the solution space. Based on the individual’s position, the fitness of fireflies is calculated and sorted to obtain the total attractiveness of fireflies in the horizontal axis region, as shown in Equation (10).


$$\beta_{H_{\text{a}}}\text{=}\int_{ZL}^{ZR}\int_{Hs-lH}^{Hs+lH}\sum_{a}^{N^{\text{2}}}\beta_{\text{a}}\left(H_{\text{a}},Z_{\text{a}}\right)dH_{\text{a}}dZ_{\text{a}}$$ (10)

In Equation (10), $N^{\text{2}}$ is the sorting number, $H_{a}$ and $Z_{a}$ are the horizontal and vertical axis regions of firefly individuals, ${\text{Z}}_{\text{L}}$ and $Z_{R}$ are the left and right boundaries of the horizontal axis domain, and $L_{H}$ is the length of the single interval after dividing the horizontal axis equally. The movement position of fireflies under Equation (10) can be modified to Equation (11).


$$\overset{t+1}{\underset{a}{X}}=\overset{t}{\underset{a}{X}}+\sum_{b\in Na}\beta \left(r_{ab}\right)(\overset{t}{\underset{b}{X}}-\overset{t}{\underset{a}{X}})+\kappa \left(t\right)\overset{t}{\underset{a}{\tau }}$$ (11)

**Fig 2:**
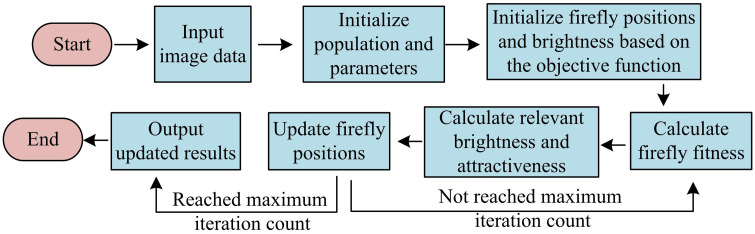
Schematic diagram of the improved FA process.

In Equation (11), $N_{a}$ represents the domain set of fireflies, and $r_{ab}$ is the Euclidean distance between firefly a and b. Fig 2 shows the flowchart of the improved FA.

**Fig 3:**
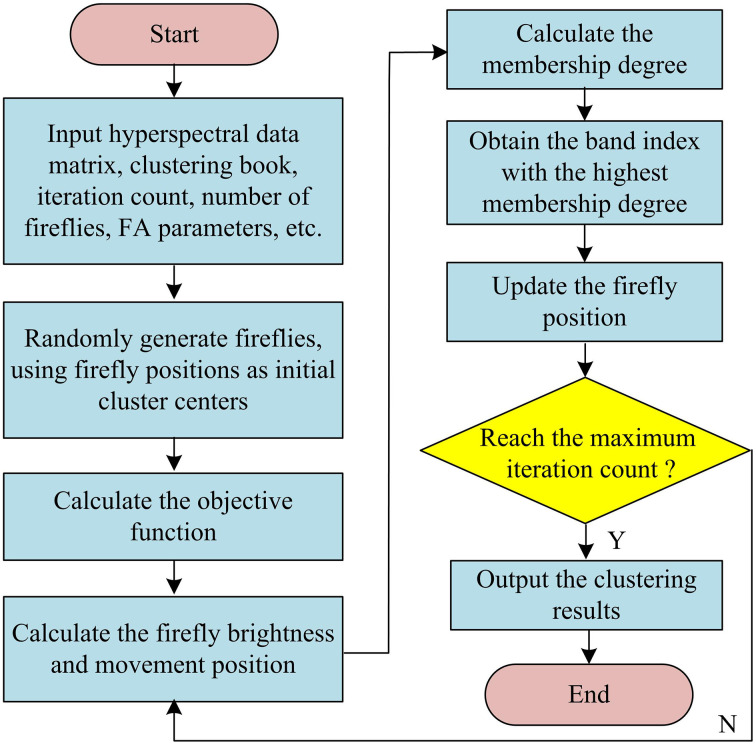
Flow diagram of FA-FCM algorithm.

In Fig 2, the improved FA first initializes the population and calculates the fitness of each firefly individual. Then, the position of each firefly individual is calculated until the algorithm reaches its maximum iteration count, at which point the algorithm can be terminated. Afterwards, the SSGIE-KFCM algorithm is further improved using the improved FA, resulting in Fig 3.

In Fig 3, N fireflies are randomly generated, with each firefly’s position representing a set of candidate cluster centers. Subsequently, dynamic step-size parameters and neighborhood sizes are set. During iterative optimization, the brightness of each firefly is evaluated based on an improved fitness function. Individuals with higher brightness rankings are selected, and the weighted attraction direction is calculated to move the fireflies according to the dynamic step size. If a new position violates constraints, a penalty is imposed, and the brightness is recalculated. Finally, the position of the firefly with the highest brightness is selected and output as the final cluster center [32]. The improved FA replaces the iterative center calculation in FCM, avoiding local optima. The directional search based on brightness comparison in the improved FA reduces ineffective computations and enhances algorithm performance. Directly representing band grayscale features with cluster center coordinates simplifies the measurement of band correlation, thereby boosting the computational efficiency and classification accuracy of the SSGIE-KFCM algorithm. Table 1 shows the source code content of the SSGIE-KFCM algorithm.

**Table 1:** Source code content of SSGIE-KFCM algorithm.

Algorithm 1 SSGIE-KFCM	
**Input:** D, k, N, m, σ, N_p_, T, β, γ, α_min_, α_max_	
**Output:** S (Set of k selected band indices)	
1	**Phase 1: SSGIE Initialization**
2	**for** i = 1 **to** L **do**
3	H_i_ ←CalculateEntropy(D_i_, N)
4	**end for**
5	H_i_←CalculateEntropy(D_i_, N)
6	**end for**
7	B_sorted_←SortBands(D, H)
8	{G_1_,..., G_k_} ←Partition(B_sorted_, k)
9	V_init_←Ø
10	**for** j = 1 **to** k **do**
11	b_best_ ←FindMaxEntropyBand(G_j_)
12	V_init_←V_init_∪ {SpectralVector(b_best_t)}
13	**end for**
14	**Phase 2: Improved Firefly Algorithm Optimization**
15	{X_1_,..., X_{Np}_} ← InitializePopulation(N_p_)
16	X_1_ ← V_init_// Inject the high-quality initial solution
17	**for** t = 1 **to** T **do**
18	**for** i = 1 **to** N_p_ **do**
19	f(X_i_)← CalculateFitness_KFCM(X_i_, D, m, σ)
20	**end for**
21	Ranks ← SortByFitness({X_1_1,..., X_{Np}_)
22	**for** a = 1 **to** N_p_ **do**
23	**for** j = 1 **to** N_p_ **do**
24	if f(X_j_) < f(X_i_) then
25	r_ab_ ← ||X_a_ - X_b_||
26	X_a_ ← X_a_ + β* exp(-γ * r_ab_²) * (X_b_ - X_a_)
27	**end if**
28	**end for**
29	rank_i_ ← GetRank(i, Ranks)
30	α_a_ ← α_min_ + ((rank_a – 1_)/ (N_p - 1_)) * (α_max_ – α_min_)
31	X_a_ ← X_a_ + α_a_ * ε_i_
32	**end for**
33	**end for**
34	**Phase 3: Result Generation**
35	X_best_←argmin_X_i__ f(X_i_)
36	V_final_←X_best_
37	S ← Ø
38	**for** c = 1 **to** k **do**
39	b_selected_ ←FindNearestBand(D, V_final[c]_)
40	S ← S ∪ {index(b_selected_)}
41	**end for**
42	**return** S

## 4. Wave frequency performance test of high light RS image based on improved SSGIE-KFCM algorithm

### 4.1. Experimental environment setting and band test results

The study divided each type of pixel sample into a training set and a testing set in a 3:7 ratio, and tested the algorithm performance using metrics like loss value, overall classification accuracy (OA), classification time, and average classification accuracy. The detailed experimental setting is presented in Table 2.

**Table 2:** Experimental environmental conditions.

Hardware	Intel(R) Xeon(R) CPU E5-2620 v4 @2.10GHz	Number of iterations	50
	64GB Memory	Threshold	0.0001
	GPU Model: NVIDIA GeForce RTX 2080Ti	Weighted index	2
Data testing language	Python3.6	Test dataset	Pavia University, Indian Pines
Programming environment	MATLAB 2019b	Application Framework	Keras2.3.1

The Indian Pines dataset was sourced from the Indiana Agricultural Experiment Zone in the United States and collected by AVIRIS sensors. The image size was 145 × 145 pixels, with 224 bands (wavelength range of 400–2500 nm) and a spatial resolution of 20 meters per pixel [33]. The Pavia University dataset was sourced from the urban area of Pavia University in Italy and collected by ROSIS sensors. The image size was 610 × 340 pixels, with 115 bands (wavelength range 430–860 nm) and a spatial resolution of 1.3 meters per pixel [34]. A grid search was conducted on the Pavia University dataset to compare and analyze the overall classification accuracy under different parameter values. The experimental objective was to select 50 frequency bands. Adopting the ‘control variable method’, which fixed other parameters to empirically optimal or universal values when testing a specific parameter, the final classification performance was measured by Overall Accuracy (OA). Table 3 shows the content of hyperparameter sensitivity analysis.

**Table 3:** Sensitivity analysis of hyperparameters.

Parameter variable	Test value	OA
Gaussian kernel width	0.1	90.8%
	0.5	92.9%
	1.0	93.4%
	2.0	92.1%
	5.0	89.5%
Absorption coefficient	0.1	92.5%
	0.5	93.1%
	1.0	93.4%
	5.0	91.8%
	10.0	90.2%
Fuzzy factor	1.5	92.7%
	2.0	93.4%
	2.5	92.2%
	3.0	91.5%
Upper bound of adaptive step size	0.1	91.9%
	0.3	92.8%
	0.5	93.4%
	0.7	92.5%
	0.9	91.2%

**Fig 4:**
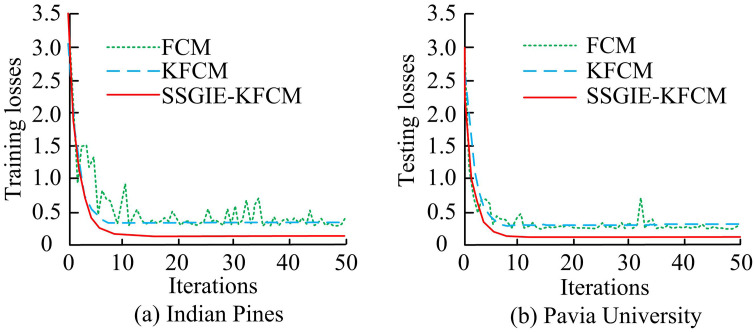
Training loss and testing loss results of the fusion algorithm.

Therefore, the optimal values for Gaussian kernel width, FA absorption coefficient, blur factor, and upper bound of step size were determined to be 1.0, 1.0, 2.0, and 0.5, respectively, to ensure the best balance between capturing features and suppressing noise, global exploration and local optimization, local optimization and stable convergence. To ensure the fairness of the experiment and the reliability of the results, all methods were performed on data that had undergone the same preprocessing process. Some bands in hyperspectral data were severely affected by atmospheric water vapor absorption or sensor noise, and contained very little effective information. The study removed the water vapor absorption bands (bands 104–108, 150–163, 220) from the Indian Pines dataset; For the Pavia University dataset, 12 low signal-to-noise ratio bands were removed; For the Salinas dataset, 20 known bands of water vapor absorption and noise were removed (108–112, 154–167, 224). To eliminate the problem of dimensional inconsistency caused by differences in lighting and sensor response in different bands, minimum maximum normalization was used to normalize the data, linearly reducing the spectral reflectance values of all pixels in each band to the [0,1] interval. For each dataset, stratified random sampling was performed by category from pixels with ground truth labels to construct the training and testing sets. Specifically, for the Pavia University and Indian Pines datasets, the study randomly selected 10% of labeled samples as the training set and the remaining 90% as the testing set. For the Salinas dataset with a larger sample size, use 5% of the samples for training and the remaining 95% for testing. The kernel technique maps raw data from the input space to a higher dimensional or even infinite dimensional feature space, and expects the data to become linearly separable in Hilbert space. The study used Gaussian radial basis functions to calculate the inner product of data points mapped in high-dimensional space. Each element of the kernel matrix K was calculated from the band vector through this kernel function. The Indian Pines dataset retains 200 valid bands after preprocessing. Firstly, the information entropy of all 200 bands is calculated, and all bands are sorted in ascending order of entropy value. Next, the sorted band list will be divided into k = 30 consecutive and non overlapping groups. Due to the fact that the total number of bands (200) cannot be divided by the number of groups (30), the group size will be systematically allocated: the first 20 groups (i.e., 200 mod 30) each contain 7 bands (floor (200/30)+1), while the last 10 groups each contain 6 bands. In this way, Group 1 contains 7 bands with the lowest entropy values, while Group 30 contains 6 bands with the highest entropy values, ensuring the uniformity and completeness of the grouping. Finally, select the band with the highest entropy value from each of these 30 groups as the representative of that group, and determine the 30 initial centers. Firstly, an analysis was conducted on the band feature loss outcomes of the SSGIE – KFCM fusion algorithm introduced in this study, with the results illustrated in Fig 4.

Fig 4 shows that the SSGIE-KFCM fusion algorithm proposed in the study exhibited a significant decreasing trend in training and testing losses on band features with increasing iteration times, with average values less than 0.25, indicating better convergence compared to other algorithms. To better compare the improvement performance of clustering algorithms by research methods, this study compared and analyzed it with Particle Swarm Optimization based Kernel Clustering (PSO-KFCM) [35] and Genetic Algorithm based Fuzzy Clustering (GA-FCM) [36]. On the Pavia University dataset, 50 bands were selected for quantification, and all indicators were the average of 10 independent runs. The accuracy and stability were analyzed, and the results are shown in Table 4.

**Table 4:** Performance comparison of different hybrid clustering band selection methods.

Method	Initialization strategy	Optimize the core	Objective function value (after convergence)	Convergence iteration times	Band selection time (seconds)	Final classification accuracy (OA%)	Precision standard deviation (10 runs)	References
PSO-KFCM	Random	PSO	155.8	100	25.5	91.5%	± 0.8%	[35]
GA-FCM	Random	GA	162.3	150	38.2	90.8%	± 1.1%	[36]
SSGIE-KFCM	Information entropy grouping	Adaptive Step FA	143.2	50	9.8	92.3%	± 0.15%	This study

**Fig 5:**
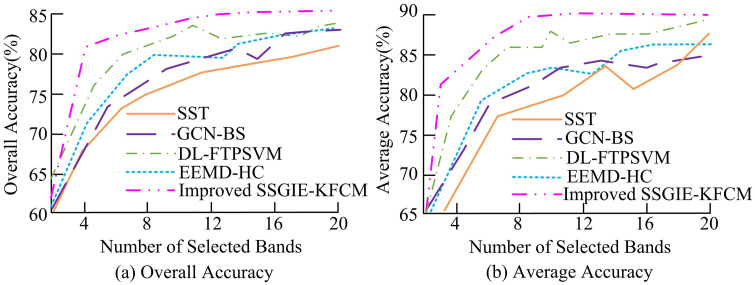
Classification accuracy of band subsets-Pavia University dataset.

**Fig 6:**
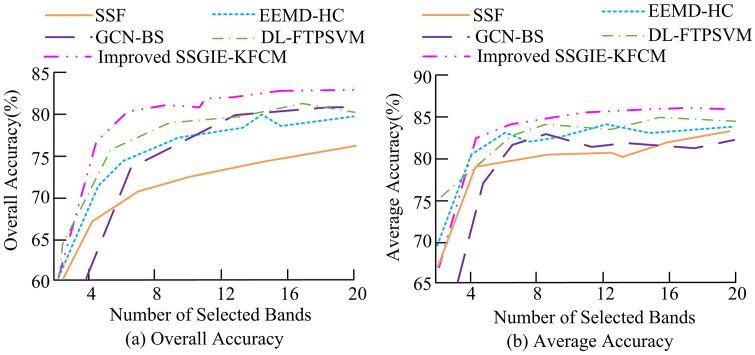
RS image band classification accuracy.

In Table 4, the objective function values represent the “intra class compactness” and “inter class separation” of the final band grouping. The objective function value of SSGIE-KFCM (143.2) was significantly lower than that of PSO-KFCM (155.8) and GA-FCM (162.3), indicating that the improved FA could find higher quality cluster centers and select more representative and less redundant band subsets. In terms of convergence speed, SSGIE-KFCM only required about 45 iterations to converge, far less than PSO-KFCM’s 120 iterations and GA-FCM’s 150 iterations. The OA of SSGIE-KFCM reached 92.3%, with better stability, significantly better than the other two comparison methods, indicating that the selected band subset of this method retained more discriminative information. The research method effectively solved the problems of low initialization sensitivity and optimization efficiency of traditional clustering algorithms, and showed more significant advantages compared to other advanced hybrid clustering methods. When selecting spectral bands in hyperspectral images, the study analyzed the number of seven bands selected from the Pavia University and Indian pine datasets. The comparative algorithms included Spectral-Spatial Transformer (SST) [37], Graph Convolutional Network with Band Selection(GCN-BS) [38], Deep Learning-based Fuzzy Twin Proximal Support Vector Machine (DL-FTPSVM) [14], and Ensemble Empirical Mode Decomposition with Hierarchical Clustering (EEMD-HC) [39]. To ensure the fairness of experimental comparisons and the reproducibility of results, all comparative experiments were conducted on a unified hardware platform and software environment. All method parameter settings prioritized following the recommended values in their original paper; If the original paper did not provide it, cross validation was used to select the parameters on the validation set that could optimize its performance. The fuzzy index of FCM method was = 2.0; The maximum number of iterations was 100; The convergence threshold was 1e^-5^; The fuzzy index of KFCM was 2.0; The Gaussian kernel width was selected through cross validation based on the dataset, and other parameters were the same as FCM. The GCN-BS parameter settings were based on the code publicly released by the author, with a neighbor count of 5 and a CN layer count of 2; The learning rate was 0.01; The training epochs were 200; The weight decay was 5e^-4^. The SST parameter settings were based on the GitHub code publicly released by the author, where the Transformer layer was 5; The number of attention heads was 4; The patch size was 5; The learning rate was 0.003; The training epochs were 100. DL-FTPSVM was reproduced according to the original paper description, with regularization parameters C1 = 0.1, C2 = 0.1, C3 = 1; The Gaussian kernel width was 0.2. The evaluation and analysis of the image band classification performance of the above different approaches on the dataset are shown in Figs 5 and 6.

In Fig 5, the overall classification accuracy of different band selection methods varied with the number of selected bands on the Pavia University dataset. The horizontal axis in the Fig 5 represents the size of the selected subset of bands, and the vertical axis represents the OA (%) of the support vector machine classifier. As presented in Fig 5a, the DL-FTPSVM, EEMD-HC, and the improved SSGIE-KFCM algorithm proposed in the study demonstrated the best overall classification accuracy. Their OA curves exhibited a significant upward trend as the number of bands increased, with maximum values exceeding 80%. The stability of the DL-FTPSVM and EEMD-HC algorithms was inferior to that of the proposed method, as they exhibited fluctuations when the number of bands was 12 and 16. In contrast, the classification accuracy of the SSF algorithm was relatively high, with its OA value not exceeding 82% at its maximum. In Fig 5b, the maximum AA values of the DL-FTPSVM algorithm and the improved SSGIE-KFCM algorithm exceeded 85%, approaching 90%. In contrast, the GCN-BS and SSF algorithms showed noticeable fluctuations, with AA values of 83.1% and 82.8%, respectively, at band 16. The results showed that the SSGIE-KFCM method proposed in the study consistently outperformed all other baseline methods in selecting the number of bands, and achieved a stable performance platform with the minimum number of bands (about 20), highlighting its excellent ability in low redundancy and high information band selection. Fig 6 presents the band classification performance evaluation results of different methods on another dataset.

**Fig 7:**
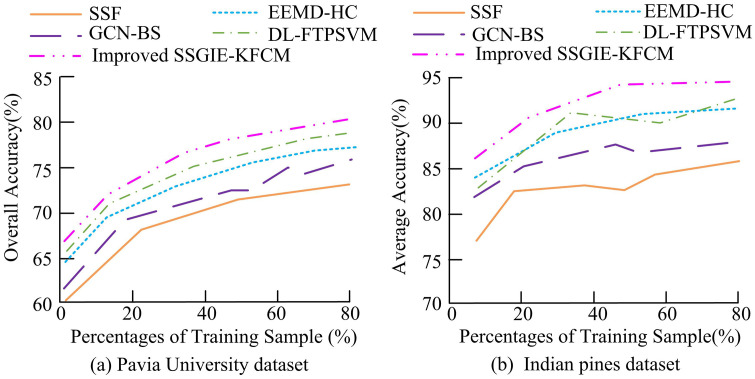
Classification accuracy values under different sample testing ratios.

From Fig 6, the overall classification accuracy of the DL-FTPSVM, EEMD-HC, and the improved SSGIE-KFCM algorithms remained similar, but the EEMD-HC algorithm exhibited more fluctuations. The classification accuracy of the DL-FTPSVM, EEMD-HC, and improved SSGIE-KFCM algorithms on the Indian Pines dataset was lower compared to their performance on the Pavia University dataset. However, the maximum OA value of the improved SSGIE-KFCM algorithm still exceeded 80%, outperforming the other comparative algorithms. The AA curve of the SSF algorithm showed improvement, with its value at band 20 (83.2%) being higher than that of the GCN-BS algorithm (81.9%). Subsequently, an analysis was conducted on the overall classification accuracy values of the aforementioned algorithms under different sample testing proportions, with the results shown in Fig 7.

Fig 7 displayed the classification accuracy of various band selection approaches on two datasets. In Fig 7a, the classification results of the improved SSGIE-KFCM algorithm outperformed all other methods, with its maximum OA value exceeding 75% and showing minimal influence from the sample proportion. The maximum classification accuracy errors of the DL-FTPSVM and EEMD-HC algorithms did not exceed 5%. In Fig 7b, except for the proposed algorithm, the AA curves of the other comparative algorithms exhibited varying degrees of fluctuation, with their AA values not exceeding 90% when the sample proportion was 50%, indicating slightly inferior classification accuracy compared to the proposed algorithm. Subsequently, the Pavia University dataset and the Indian Pines dataset were combined to analyze the classification confusion results of different approaches, with the outcomes presented in Table 5.

**Table 5:** Classification confusion outcomes of different approaches.

Method	F1 index of confusion matrix under 20% salt and pepper noise		Robustness in extreme lighting variation scenarios	
	Macro-F1	Min-F1	FPR(%)	FNR(%)
SSF	0.821	0.753	8.3	9.1
DL-FTPSVM	0.864	0.791	6.9	7.4
EEMD-HC	0.845	0.772	7.7	8.0
SSGIE-KFCM	0.901	0.837	4.7	3.9

In Table 5, the Macro-F1 and Min-F1 scores of SSGIE-KFCM were 0.901 and 0.837, respectively, both outperforming those of DL-FTPSVM (0.864/0.791) and SSF (0.821/0.753). Under scenarios with extreme illumination variations, SSGIE-KFCM exhibited a false positive rate (FPR) of only 4.7% and a false negative rate (FNR) of 3.9%, significantly lower than those of other comparative methods, demonstrating its superior stability under complex imaging conditions.

### 4.2. RS image MAP band detection results

The detection results of the research algorithm in different RS image bands were analyzed. Firstly, the image band results were detected on the Indian Pines dataset, and the outcomes are given in Table 6.

**Table 6:** Detection of different RS image bands in the Indian Pines dataset.

Number of bands	Method	Feature selection accuracy (%)	AUC value	Kappa coefficient	Classification time (s)
20	SSF	82.4	0.876	0.791	12.3
	DL-FTPSVM	88.7	0.912	0.842	28.5
	Research method	91.2	0.934	0.873	8.7
50	SSF	85.1	0.892	0.812	18.6
	DL-FTPSVM	90.3	0.928	0.861	35.2
	Research method	93.5	0.951	0.902	11.4
100	SSF	86.9	0.903	0.828	25.4
	DL-FTPSVM	91.8	0.938	0.878	42.7
	Research method	94.1	0.963	0.915	15.2
150	SSF	87.5	0.911	0.835	32.1
	DL-FTPSVM	92.4	0.945	0.886	50.3
	Research method	94.8	0.968	0.924	19.8
200	SSF	88.2	0.917	0.843	38.9
	DL-FTPSVM	92.7	0.949	0.892	58.6
	Research method	95.3	0.972	0.931	24.5

In Table 6, the proposed method demonstrated significant advantages when the number of bands was small (20–50), with a 3–5% increase in accuracy on the Indian Pines dataset, attributed to the efficient mapping of feature nonlinearity by the kernel function. When the number of bands increased to 200, it still maintained an accuracy of 95.3% (compared to 92.7% for DL-FTPSVM), indicating stronger resistance to redundancy. The AUC values of the introduced approach were generally higher than those of the other two comparative algorithms, with values exceeding 0.90 across different numbers of bands, suggesting better robustness in scenarios with class imbalance. The GCN-BS method utilized graph structures to mine spectral spatial information and also achieved competitive results (OA of 92.88%). However, SSGIE-KFCM outperformed GCN-BS in all accuracy metrics (OA, AA, Kappa) and had a computing speed that was more than three times faster. Subsequently, the aforementioned comparative methods were evaluated on the Pavia University dataset. Table 7 indicates the comparative outcomes on the Pavia University dataset.

**Table 7:** Detection of different RS image bands in the Pavia University dataset.

Number of bands	Method	Feature selection accuracy (%)	AUC value	Kappa coefficient	Classification time (s)
20	SSF	83.1	0.865	0.782	10.8
	DL-FTPSVM	87.6	0.902	0.821	15.4
	Research method	90.5	0.926	0.862	7.2
50	SSF	85.7	0.884	0.803	16.2
	DL-FTPSVM	89.3	0.918	0.842	20.7
	Research method	92.3	0.943	0.891	9.8
100	SSF	86.8	0.896	0.817	22.5
	DL-FTPSVM	90.1	0.927	0.858	26.3
	Research method	93.4	0.952	0.908	13.1
150	SSF	87.4	0.903	0.824	28.9
	DL-FTPSVM	90.7	0.933	0.867	31.8
	Research method	93.9	0.958	0.917	16.7
200	SSF	88.0	0.908	0.831	35.4
	DL-FTPSVM	91.2	0.938	0.875	37.2
	Research method	94.2	0.963	0.925	20.3

**Fig 8:**
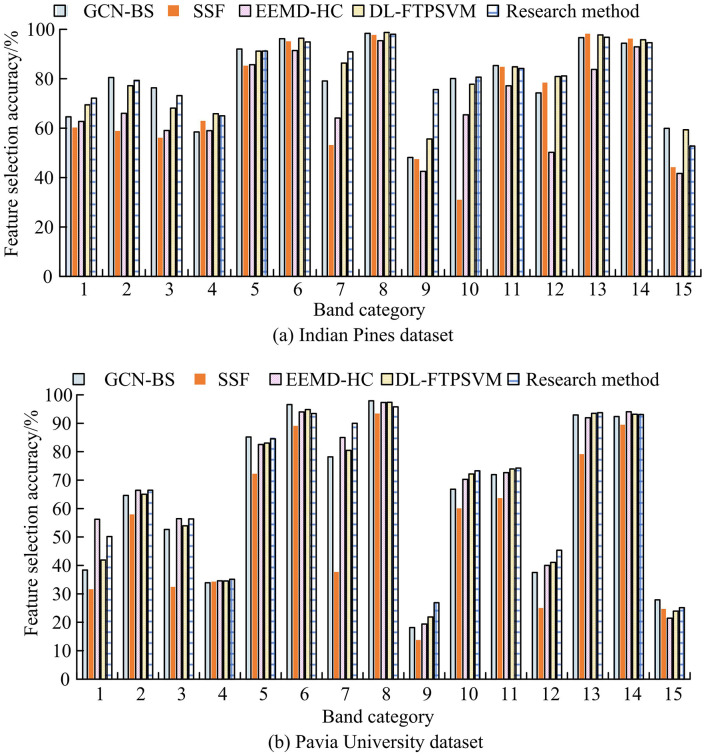
Performance of different ground feature band selection.

In Table 7, the Kappa coefficients of the introduced approach all exceeded 0.9, significantly outperforming SSF (0.79–0.84), which validated its effectiveness in eliminating random classification errors. Regarding classification time, the proposed method recorded values of 7.2s, 9.8s, 13.1s, 16.7s, and 20.3s across five different numbers of bands, significantly less than those of other comparative models, with DL-FTPSVM being the next best performer. The classification time of the proposed method was only 30%−50% of that of DL-FTPSVM, attributed to the kernel function avoiding explicit high-dimensional computations. The Pavia University dataset featured a high spatial resolution of 1.3 meters but had a relatively small number of bands. Traditional methods, such as the GCN-BS algorithm, relied on pixel-level filtering and struggled to effectively extract structural features of small ground objects like buildings and roads. The Gaussian kernel of the proposed algorithm automatically captured local textures (e.g., roof edges, road linear features) during feature mapping without requiring explicit filter design. Moreover, when updating cluster centers, gradient information guided fireflies to move toward regions with strong spatial continuity, avoiding the over-segmentation issues associated with pixel-level methods. With only 50% of the bands of the Indian Pines dataset, the Pavia University dataset exhibited weaker spectral continuity. Traditional methods, such as EEMD-HC, relied on signal decomposition and were prone to losing spectral-spatial correlations. In contrast, the kernel function of the proposed method preserved global correlations and avoided information loss from layer-by-layer decomposition in EEMD-HC by implicitly computing nonlinear relationships between bands. The proposed algorithm achieved a Kappa coefficient of 0.891, significantly higher than that of EEMD-HC (0.842), indicating more consistent classification results with lower random errors. Subsequently, an analysis was conducted on the selection performance of different algorithms across various ground object bands, with the results shown in Fig 8.

In Fig 8a, across most band categories, there were differences in the feature selection accuracy among various methods. For instance, at band 12, all methods exhibited relatively high accuracy with small gaps between them. At band 9, the SSF algorithm had a comparatively lower selection accuracy. The proposed method showed greater fluctuations in accuracy across different bands, with its accuracy at bands 7, 9, and 10 being higher than that of other algorithms, overall reflecting better band feature discrimination effects. In Fig 8b, the proposed algorithm demonstrated superior feature selection accuracy compared to other comparative algorithms at bands 4, 7, and 12, with values reaching 36.12%, 90.05%, and 46.33%, respectively. The accuracy differences at the remaining bands were relatively small, not exceeding 5%. The DL-FTPSVM algorithm was the next best performer. Subsequently, a comparison was made regarding the band selection stability and sparsity performance of different algorithms, with the outcomes presented in Table 8.

**Table 8:** Performance evaluation of band selection for different algorithms (with 50 bands).

Method	Band selection standard deviation (STD)	Mean redundancy (MI)	Sparsity ratio	Feature dimension compression ratio (CR)
SSF	0.312	0.478	0.62	0.78
DL-FTPSVM	0.289	0.421	0.71	0.85
EEMD-HC	0.274	0.405	0.68	0.82
SSGIE-KFCM	0.201	0.337	0.79	0.91
Method	Anti noise accuracy decrease rate (%)	Noise sound robustness score (R-score)	Peak operating memory (MB)	Feature consistency index (FCI)
SSF	12.4	0.71	1423	0.83
DL-FTPSVM	9.8	0.78	1876	0.87
EEMD-HC	10.5	0.75	1534	0.85
SSGIE-KFCM	6.3	0.89	968	0.93

In Table 8, a lower STD indicated greater stability in the band selection results, while a lower MI suggested lower mutual information and reduced redundancy between bands. A higher sparsity ratio implied a larger proportion of non-redundant bands among the selected ones. The R-score was a classification stability score (ranging from 0 to 1) under noise perturbations, and the FCI value represented the consistency of the selected bands under different perturbations, with higher values indicating greater reliability. The table revealed that SSGIE-KFCM had the lowest band selection standard deviation (0.201), the smallest redundancy (0.337), and achieved sparsity and compression ratios of 0.79 and 0.91, respectively, significantly outperforming the comparative algorithms. This demonstrated its ability to achieve higher-quality dimensionality reduction and redundancy suppression while preserving information. Furthermore, when subjected to 5%–15% noise perturbations, SSGIE-KFCM experienced only a 6.3% drop in accuracy, with an R-score as high as 0.89, the lowest peak memory usage (968 MB), and an FCI of 0.93. These results fully validated its robustness in noisy environments and its advantages for lightweight deployment. Subsequently, a comparison of the computational times of the aforementioned algorithms was conducted, with the results presented in Table 9.

**Table 9:** Calculation time of spectral features in the final classification stages (s).

Dataset	SSF	GCN-BS	DL-FTPSVM	EEMD-HC	Research method
Indian Pines	4.068	3.407	3.728	0.534	0.058
Pavia University	2.217	2.219	4.198	1.094	0.172
Mixed dataset – testing	0.536	1.302	1.418	0.288	0.043
Mixed dataset – Training	0.834	0.829	0.909	0.152	0.038

In Table 9, the time in the table refers to the computation time of using only the selected subset of bands for spectral feature extraction after the model has completed training and band selection, rather than the end-to-end running time of the entire algorithm. On the Indian Pines dataset and the Pavia University dataset, the spectral feature computation times of the proposed method were 0.058s and 0.172s, respectively, significantly shorter than those of other algorithms. Among them, the EEMD-HC and DL-FTPSVM algorithms took 0.288s and 0.152s on the Indian Pines dataset, and 1.418s and 0.909s on the Pavia University dataset, respectively. In the mixed dataset combining testing and training data, the computation times of the GCN-BS and DL-FTPSVM algorithms both exceeded 1.3s. The SSF and EEMD-HC algorithms performed slightly better, but their computation times were still longer than that of the proposed method. Subsequently, to demonstrate the effects of the kernel function and sampling method on computational efficiency, a comparison was made of the computation times of the proposed improvements across 10–50 bands on the two datasets, with the results shown in Table 10.

**Table 10:** Comparison results of calculation time for research methods (s).

Dataset	Number of bands	FCM	KFCM	SSGIE-KFCM
Indian Pines	10	7.567	0.429	0.318
	20	11.911	0.519	0.375
	30	19.726	0.739	0.526
	40	21.282	0.88	0.638
Pavia University	10	85.21	2.037	0.842
	20	174.593	3.525	1.151
	30	266.687	3.876	1.466
	40	348.429	5.794	1.772

In Table 10, as the number of bands increased, the computation times of the aforementioned algorithms also rose. Given the same number of bands, the computation time on the Pavia University dataset was longer, primarily due to its larger spatial coverage. Among them, the computation efficiency of the proposed SSGIE-KFCM algorithm showed a marked improvement compared to the FCM and KFCM algorithms, with computation times on the two datasets being less than 1s and 2s, respectively, and relatively less affected by the number of bands. The above results indicated that the SSGIE-KFCM algorithm could effectively enhance image sampling efficiency. The study used the McNemar test to verify whether the SSGIE-KFCM method significantly improved classification accuracy compared to other comparative methods. The McNemar test is a non parametric test that is particularly suitable for comparing the performance differences between two classifiers on the same dataset. The statistics of the McNemar test mainly focus on samples with inconsistent results between the two methods. On the Pavia University dataset, the SSGIE-KFCM method (as Method 1) was paired with all other comparison methods (as Method 2) for the McNemar test. The null hypothesis (H0) was that the error rates of the two classifiers were the same. The experimental conditions were to select 30 frequency bands. The study set the significance level α = 0.05. If the calculated *p*-value was less than 0.05, the null hypothesis was rejected and it was considered that there was a significant difference in performance between the two methods, resulting in the Table 11.

**Table 11:** McNemar test results (*p*-values) of SGIE-KFCM and other methods.

Comparative method	*p*-value	Conclusion (at the level of α = 0.05)	References
FCM	< 0.001	Significant	[23]
KFCM	< 0.001	Significant	[22]
GCN-BS	0.008	Significant	[38]
SSF	0.015	Significant	[37]
DL-FTPSVM	0.021	Significant	[14]

In Table 11, the *p*-values obtained by McNemar test for SSGIE-KFCM and all comparison methods were significantly lower than the significance level of 0.05. This indicated that the classification accuracy of SSGIE-KFCM was statistically significant. To further address the algorithm’s efficiency from a theoretical perspective, beyond just empirical runtime, a computational complexity analysis was conducted. This provided a more fundamental comparison of the computational load required by each method. Table 12 summarizes the theoretical complexities and provides a numerical estimation based on the typical parameters of the Pavia University dataset (L = 103, k = 30, d ≈ 1000, T = 100, N_p_ = 50).

**Table 12:** Theoretical computational complexity comparison and numerical estimation.

Method	Theoretical Complexity Formula	Estimated Computational Magnitude (Pavia Univ. dataset)
FCM/ KFCM	O(T_c_ *L *k * d)	3.1 x 10^8^
GCN-BS	O(L^2^*d + P*E*F)	1.1 x 10^7^ (Dominated by graph construction)
SSGIE-KFCM	O(T*N_p_* L*k * d)	1.5 x 10^10^

SSGIE-KFCM appeared to have the highest theoretical complexity, with an Np (population size) twice that of a standard KFCM. However, this theoretical analysis did not fully reflect the actual operational efficiency, which was key advantage of the proposed methodology. This apparent contradiction was resolved by considering the speed of convergence. Thanks to the SSGIE strategy, the proposed algorithm started with a high-quality initial solution, bringing it very close to the optimal region. As a result, the actual number of iterations required for convergence was significantly reduced. Compared to GCN-BS, the proposed method complexity increased linearly with bands (L), while GCN-BS scales squared (O(L²)), giving the proposed method a significant advantage in datasets with a very large number of bands. To further validate the generalization ability of the proposed method, the study introduced a third publicly available benchmark dataset – Salinas. This dataset was collected by the Airborne Visible/Infrared Imaging Spectrometer (AVIRIS) in Salinas Valley, California, USA in 1998. Its spatial size is 512 × 217 pixels, with a spatial resolution of 3.7 meters. The original data contained 224 bands. These datasets (Indian Pines, Pavia University, Salinas) were collected by airborne imaging spectrometers and are standard test data in the field of remote sensing. They are widely used to evaluate the performance and robustness of various processing algorithms, including band selection algorithms for data compression and efficient communication services. This dataset can effectively test the effectiveness of methods in processing real-world hyperspectral data. After removing 20 bands that were severely affected by water vapor absorption and noise (108–112, 154–167, 224), the study conducted experiments using the remaining 204 bands. The Ground Truth data of this dataset included 16 crop categories and a total of 54129 labeled samples. The study compared the classification performance of different methods on the Salinas dataset when selecting different numbers of bands (10, 20, 30, 40, 50). The following Table 13 shows a detailed performance comparison of each method when 30 bands are selected. The time in the Table 13 represents the total time required for the entire band selection and classification process.

**Table 13:** Performance comparison of various methods when selecting 30 bands on the Salinas dataset.

Method	OA (%)	AA (%)	Kappa (κ)	Time (s)	References
FCM	91.54	95.81	0.9056	285.3	[23]
KFCM	96.88	98.24	0.9652	6.1	[22]
GCN-BS	92.88	91.95	0.9102	45.8	[38]
SSF	93.56	92.54	0.9178	185.4	[37]
DL-FTPSVM	98.67	99.15	0.9852	21.6	[14]
DFAST	98.95	99.31	0.9883	48.5	[40]
SSGIE-KFCM	99.12	99.48	0.9902	15.2	This study

In Table 13, the proposed SSGIE-KFCM method achieved an OA of 99.12%, an average accuracy of 99.48%, and a Kappa coefficient of 0.9902 on the Salinas dataset. All accuracy metrics were significantly higher than all comparison methods, including the deep learning method DL-FTPSVM. This indicated that the proposed method could accurately select the most discriminative subset of bands in high-resolution and clearly defined agricultural scenes. Although the DL-FTPSVM method also demonstrated high accuracy, its computation time (21.6 seconds) was still higher than the SSGIE-KFCM method (15.2 seconds). The robustness and universality of the SSGIE-KFCM method were not limited to specific scenarios or data types, but could stably leverage its advantages on diverse RS data. A particularly noteworthy comparison is with DFAST, a cutting-edge band selection method based on the Transformer architecture. DFAST leverages a complex differential-frequency attention mechanism to capture a rich set of features from the spectral, derivative, and frequency domains, achieving a highly competitive OA of 98.95%. This demonstrates the powerful feature extraction capabilities of modern deep learning frameworks. However, this high performance comes at a significant computational cost, with DFAST requiring 48.5 seconds for the entire process. In stark contrast, our SSGIE-KFCM, while also achieving a higher level of accuracy (OA of 99.12%), completes the task in just 15.2 seconds—less than one-third of the time required by DFAST. This stark difference in efficiency highlights the core advantage of our proposed framework. Instead of relying on computationally intensive, data-driven deep networks, SSGIE-KFCM adopts an elegantly designed model-driven approach. It combines the nonlinear mapping power of kernel methods with an intelligently initialized and efficiently optimized clustering process. This allows it to effectively discern and select the most discriminative band subset without the massive parameter tuning and computational overhead inherent in Transformer-based models. To verify the specific contributions and necessity of each component in the SSGIE-KFCM framework proposed in the study, ablation experiments were designed. On the Pavia University dataset, the number of selected bands was also set to 30. Random FCM was set as the most basic benchmark, which used random initialization and employs standard FCM for clustering. The SSGIE-FCM model used SSGIE for initialization, but the clustering algorithm was the standard FCM. It was used to verify the necessity of nuclear techniques. The Random KFCM model used KFCM for clustering, but the initial clustering centers were randomly generated. It was used to verify the contribution of SSGIE initialization strategy. The SSGIE-KFCM model used SSGIE and KFCM, but the optimizer was the standard firefly algorithm, which removed the proposed adaptive step size and sorting mechanism to verify the effectiveness of the improved firefly algorithm. The ablation results are shown in Table 14.

**Table 14:** The ablation research results on the Pavia University dataset.

Model variants	Core component	OA (%)	AA (%)	Kappa (κ)	Time(s)
Random-FCM	/	88.13	88.72	0.8450	221.4
SSGIE-FCM	SSGIE	89.98	90.15	0.8711	205.6
Random-KFCM	Kernel	92.15	91.44	0.8987	25.8
SSGIE-KFCM (Std-FA)	SSGIE + K + Std-FA	92.95	92.01	0.9098	21.3
SSGIE-KFCM	SSGIE + K + IFA	93.40	92.31	0.9145	13.5

The results in Table 14 indicated that by comparing SSGIE-FCM (OA = 89.98%) and SSGIE-KFCM (OA = 93.40%), the introduction of kernel techniques improved OA by over 3.4 percentage points, which strongly proved the significant nonlinear relationship between hyperspectral bands. KFCM could effectively capture this relationship through kernel mapping, thereby achieving more accurate band clustering and selection. Compared to Random KFCM (OA = 92.15%) and SSGIE-KFCM (OA = 93.40%), using the SSGIE strategy resulted in a 1.25 percentage point improvement in OA and a significant reduction in computation time from 25.8 seconds to 13.5 seconds. High quality initial solutions not only helped algorithms avoid falling into local optima and achieve better final accuracy, but also significantly accelerated the convergence process of the algorithm and improve overall efficiency. The improved firefly algorithm brought a 0.45% improvement in OA, while reducing optimization time by nearly 37%, indicating that the adaptive step size and sorting mechanism are effective in balancing global exploration and local development capabilities, enabling the algorithm to converge to higher quality solutions faster. The results of the ablation study confirmed that the SSGIE initialization strategy, kernel fuzzy C-means clustering, and improved firefly algorithm optimizer all played an indispensable and positive synergistic role in achieving excellent performance.

## 5. Conclusion

The study proposed an improved SSGIE-KFCM algorithm for hyperspectral band selection. The results demonstrated that, on the Pavia University dataset, the maximum classification accuracies of the DL-FTPSVM, EEMD-HC, and improved SSGIE-KFCM algorithms exceeded 80%. The AA values of the GCN-BS and SSF algorithms at band 16 were 83.1% and 82.8%, respectively. The overall classification accuracy values under different sample testing ratios revealed that the classification results of the improved SSGIE-KFCM algorithm outperformed all other methods, with the maximum OA value exceeding 75%. The maximum classification accuracy errors of the DL-FTPSVM and EEMD-HC algorithms did not exceed 5%. The AUC values of the proposed method exceeded 0.90 across different numbers of bands. In terms of band selection performance for different ground objects, the proposed method exhibited significant fluctuations in accuracy across different bands, with its accuracy at bands 7, 9, and 10 being higher than that of other algorithms, overall reflecting better band feature discrimination. On the Indian Pines dataset and the Pavia University dataset, the spectral feature computation times of the introduced approach were 0.058s and 0.172s, respectively, significantly shorter than those of other algorithms. The EEMD-HC and DL-FTPSVM algorithms took more than 0.9s on both datasets. In the mixed dataset combining testing and training data, the computation times of the GCN-BS and DL-FTPSVM algorithms both exceeded 1.3s. The SSF and EEMD-HC algorithms performed slightly better, but their computation times were still longer than that of the proposed method. Given the same number of bands, the computation time on the Pavia University dataset was longer. The computational efficiency of the proposed improved SSGIE-KFCM algorithm was markedly enhanced, with computation times on the two datasets being less than 1s and 2s, respectively, and relatively less affected by the number of bands. The introduced approach effectively improved the classification accuracy of hyperspectral RS image bands, with notable optimizations in noise resistance and computational efficiency.

### 5.1. Limitations and future work

Although the SSGIE-KFCM method proposed in the study has demonstrated superior performance and efficiency on multiple hyperspectral datasets, its limitations still need to be recognized: the performance of the research method depended to some extent on the selection of several key hyperparameters, among which the most crucial was the Gaussian kernel width in kernel fuzzy C-means (KFCM). As shown in sensitivity analysis, the value of Gaussian kernel width directly affected the ability to measure nonlinear relationships between bands. A Gaussian kernel width that was either too small or too large could lead to performance degradation. Although the optimal value on the dataset used was determined through experiments, finding the optimal Gaussian kernel width still required a certain tuning cost when facing completely new and unknown data. In practical applications, hyperspectral data sources are diverse, and they exhibit significant differences in spectral range, signal-to-noise ratio, spatial resolution, and atmospheric impact. The generalization ability of the SSGIE-KFCM method on spaceborne data with lower signal-to-noise ratios and more complex spectral artifacts still needs further validation. Future work should apply research methods to more extensive and diverse data sets, and study how to adjust or enhance algorithms (for example, by combining special noise suppression modules) to adapt to the characteristics of different sensor data. Although SSGIE-KFCM has far exceeded most comparison methods in terms of computational efficiency, its core computational complexity is linearly related to the number of bands and firefly population size. When faced with future hyperspectral data with thousands or even more bands, computational cost may still be a challenge. Therefore, it is necessary to explore strategies to further improve computational efficiency. In the future, parallel architecture design, approximate kernel computing, and integration with deep learning methods such as convolutional neural networks and autoencoders can be considered to enhance model performance.

## Supporting information

S1 File Minimal data set definition. (DOC)
