# Supplementary material for: Wave frequency selection method for hyperspectral hyperspectral remote sensing image based on SSGIE-KFCM algorithm
Source: PLoS One. 2026 Apr 17;21(4):e0343986. doi: 10.1371/journal.pone.0343986 (PMC13089737; doi:10.1371/journal.pone.0343986)
Supplement: S1 File — (DOC) [file pone.0343986.s001.doc]

**The data in Figure 4**

| (a) Indian Pines | | | | | |
| --- | --- | --- | --- | --- | --- |
| FCM | | KFCM | | SSGIE-KFCM | |
| Iterations | Training losses | Iterations | Training losses | Iterations | Training losses |
| 10 | 0.82 | 10 | 0.41 | 10 | 0.20 |
| 20 | 0.36 | 20 | 0.41 | 20 | 0.20 |
| 30 | 0.57 | 30 | 0.41 | 30 | 0.20 |
| 40 | 0.36 | 40 | 0.41 | 40 | 0.20 |
| 50 | 0.41 | 50 | 0.41 | 50 | 0.20 |
| (b) Pavia University | | | | | |
| FCM | | KFCM | | SSGIE-KFCM | |
| Iterations | Training losses | Iterations | Training losses | Iterations | Training losses |
| 10 | 0.48 | 10 | 0.32 | 10 | 0.017 |
| 20 | 0.24 | 20 | 0.32 | 20 | 0.017 |
| 30 | 0.26 | 30 | 0.32 | 30 | 0.017 |
| 40 | 0.22 | 40 | 0.32 | 40 | 0.017 |
| 50 | 0.26 | 50 | 0.32 | 50 | 0.017 |

**The data in Figure 5**

| (a) Overall Accuracy | | | | | | | | | |
| --- | --- | --- | --- | --- | --- | --- | --- | --- | --- |
| SST | | GCN-BS | | DL-FTPSVM | | EEMD-HC | | Improved SSGIE-KFCM | |
| Number of Selected Bands | Overall Accuracy(%) | Number of Selected Bands | Overall Accuracy(%) | Number of Selected Bands | Overall Accuracy(%) | Number of Selected Bands | Overall Accuracy(%) | Number of Selected Bands | Overall Accuracy(%) |
| 4 | 67.84 | 4 | 67.84 | 4 | 70.02 | 4 | 74.36 | 4 | 81.47 |
| 8 | 75.02 | 8 | 76.37 | 8 | 79.25 | 8 | 81.07 | 8 | 83.26 |
| 12 | 78.23 | 12 | 79.56 | 12 | 79.86 | 12 | 82.51 | 12 | 85.00 |
| 16 | 79.25 | 16 | 81.74 | 16 | 82.51 | 16 | 82.51 | 16 | 85.00 |
| 20 | 81.05 | 20 | 83.02 | 20 | 83.25 | 20 | 83.67 | 20 | 85.00 |
| (b) Average Accuracy | | | | | | | | | |
| SST | | GCN-BS | | DL-FTPSVM | | EEMD-HC | | Improved SSGIE-KFCM | |
| Number of Selected Bands | Overall Accuracy(%) | Number of Selected Bands | Overall Accuracy(%) | Number of Selected Bands | Overall Accuracy(%) | Number of Selected Bands | Overall Accuracy(%) | Number of Selected Bands | Overall Accuracy(%) |
| 4 | 67.24 | 4 | 72.16 | 4 | 78.69 | 4 | 73.16 | 4 | 83.12 |
| 8 | 76.39 | 8 | 77.49 | 8 | 85.34 | 8 | 82.01 | 8 | 88.25 |
| 12 | 82.14 | 12 | 82.05 | 12 | 86.21 | 12 | 82.68 | 12 | 90.00 |
| 16 | 82.14 | 16 | 82.05 | 16 | 87.25 | 16 | 86.34 | 16 | 90.00 |
| 20 | 84.25 | 20 | 84.91 | 20 | 89.47 | 20 | 86.34 | 20 | 90.00 |

**The data in Figure 6**

| (a) Overall Accuracy | | | | | | | | | |
| --- | --- | --- | --- | --- | --- | --- | --- | --- | --- |
| SST | | GCN-BS | | DL-FTPSVM | | EEMD-HC | | Improved SSGIE-KFCM | |
| Number of Selected Bands | Overall Accuracy(%) | Number of Selected Bands | Overall Accuracy(%) | Number of Selected Bands | Overall Accuracy(%) | Number of Selected Bands | Overall Accuracy(%) | Number of Selected Bands | Overall Accuracy(%) |
| 4 | 67.02 | 4 | 61.37 | 4 | 71.89 | 4 | 68.25 | 4 | 75.02 |
| 8 | 71.89 | 8 | 75.00 | 8 | 77.25 | 8 | 75.89 | 8 | 81.34 |
| 12 | 74.05 | 12 | 79.25 | 12 | 78.36 | 12 | 78.21 | 12 | 82.87 |
| 16 | 74.98 | 16 | 80.24 | 16 | 80.97 | 16 | 78.97 | 16 | 84.12 |
| 20 | 76.02 | 20 | 80.57 | 20 | 80.59 | 20 | 80.05 | 20 | 84.12 |
| (b) Average Accuracy | | | | | | | | | |
| SST | | GCN-BS | | DL-FTPSVM | | EEMD-HC | | Improved SSGIE-KFCM | |
| Number of Selected Bands | Overall Accuracy(%) | Number of Selected Bands | Overall Accuracy(%) | Number of Selected Bands | Overall Accuracy(%) | Number of Selected Bands | Overall Accuracy(%) | Number of Selected Bands | Overall Accuracy(%) |
| 4 | 77.34 | 4 | 71.25 | 4 | 77.28 | 4 | 80.57 | 4 | 83.01 |
| 8 | 80.02 | 8 | 82.16 | 8 | 83.56 | 8 | 82.15 | 8 | 84.56 |
| 12 | 80.13 | 12 | 81.23 | 12 | 83.56 | 12 | 84.23 | 12 | 86.14 |
| 16 | 82.44 | 16 | 81.77 | 16 | 85.04 | 16 | 83.56 | 16 | 86.25 |
| 20 | 84.06 | 20 | 82.53 | 20 | 84.95 | 20 | 83.98 | 20 | 86.25 |

**The data in Figure 7**

| (a) Pavia University dataset | | | | | | | | | |
| --- | --- | --- | --- | --- | --- | --- | --- | --- | --- |
| SST | | GCN-BS | | DL-FTPSVM | | EEMD-HC | | Improved SSGIE-KFCM | |
| Percentages of Training Sample (%) | Overall Accuracy(%) | Percentages of Training Sample (%) | Overall Accuracy(%) | Percentages of Training Sample (%) | Overall Accuracy(%) | Percentages of Training Sample (%) | Overall Accuracy(%) | Percentages of Training Sample (%) | Overall Accuracy(%) |
| 20 | 66.23 | 20 | 68.12 | 20 | 70.87 | 20 | 72.43 | 20 | 74.15 |
| 40 | 70.25 | 40 | 71.98 | 40 | 75.69 | 40 | 74.23 | 40 | 76.94 |
| 60 | 72.14 | 60 | 74.39 | 60 | 76.65 | 60 | 76.01 | 60 | 78.35 |
| 80 | 74.06 | 80 | 76.11 | 80 | 78.34 | 80 | 77.12 | 80 | 80.25 |
| (b) Indian pines dataset | | | | | | | | | |
| SST | | GCN-BS | | DL-FTPSVM | | EEMD-HC | | Improved SSGIE-KFCM | |
| Percentages of Training Sample (%) | Overall Accuracy(%) | Percentages of Training Sample (%) | Overall Accuracy(%) | Percentages of Training Sample (%) | Overall Accuracy(%) | Percentages of Training Sample (%) | Overall Accuracy(%) | Percentages of Training Sample (%) | Overall Accuracy(%) |
| 20 | 82.47 | 20 | 85.12 | 20 | 86.25 | 20 | 86.25 | 20 | 89.97 |
| 40 | 82.65 | 40 | 86.39 | 40 | 89.77 | 40 | 89.84 | 40 | 93.25 |
| 60 | 84.68 | 60 | 86.39 | 60 | 91.93 | 60 | 92.08 | 60 | 94.78 |
| 80 | 86.35 | 80 | 87.39 | 80 | 93.12 | 80 | 92.68 | 80 | 94.78 |

**The data in Figure 8**

| (a) Indian Pines dataset | | | | | |
| --- | --- | --- | --- | --- | --- |
| Band category | GCN-BS | SSF | EEMD-HC | DL-FTPSVM | Research method |
| 1 | 65.23 | 60.00 | 70.00 | 72.28 | 73.05 |
| 2 | 81.01 | 58.00 | 65.00 | 78.44 | 80.07 |
| 3 | 74.25 | 55.00 | 57.00 | 68.23 | 73.055 |
| 4 | 65.02 | 62.00 | 59.00 | 65.04 | 64.32 |
| 5 | 92.04 | 86.04 | 84.07 | 92.01 | 91.01 |
| 6 | 98.26 | 93.03 | 96.02 | 98.05 | 97.22 |
| 7 | 80.17 | 53.02 | 85.01 | 92.06 | 95.26 |
| 8 | 99.05 | 97.05 | 98.03 | 99.02 | 98.31 |
| 9 | 55.26 | 48.47 | 43.05 | 78.07 | 81.04 |
| 10 | 80.04 | 70.46 | 78.04 | 85.04 | 86.05 |
| 11 | 80.07 | 77.25 | 85.26 | 84.05 | 85.07 |
| 12 | 75.52 | 50.34 | 81.07 | 80.30 | 82.03 |
| 13 | 98.33 | 85.02 | 98.11 | 97.02 | 98.12 |
| 14 | 96.04 | 75.20 | 95.25 | 96.07 | 96.17 |
| 15 | 60.48 | 43.01 | 42.026 | 59.04 | 53.11 |
| (b) Pavia University dataset | | | | | |
| Band category | GCN-BS | SSF | EEMD-HC | DL-FTPSVM | Research method |
| 1 | 38.38 | 31.62 | 56.22 | 41.89 | 50.17 |
| 2 | 64.63 | 57.95 | 66.42 | 65.02 | 66.44 |
| 3 | 52.65 | 32.44 | 56.45 | 53.93 | 56.34 |
| 4 | 33.89 | 34.32 | 34.58 | 34.53 | 35.11 |
| 5 | 85.16 | 72.25 | 82.54 | 83.03 | 84.51 |
| 6 | 96.58 | 89.11 | 93.99 | 94.81 | 93.42 |
| 7 | 78.18 | 37.73 | 85.05 | 80.45 | 90.07 |
| 8 | 97.91 | 93.43 | 97.3 | 97.36 | 95.76 |
| 9 | 18.13 | 13.75 | 19.38 | 21.88 | 26.88 |
| 10 | 66.79 | 60.09 | 70.26 | 72.17 | 73.26 |
| 11 | 71.94 | 63.69 | 72.63 | 73.91 | 74.25 |
| 12 | 37.52 | 25.01 | 40.22 | 41.07 | 45.33 |
| 13 | 92.93 | 79.15 | 91.95 | 93.48 | 93.72 |
| 14 | 92.37 | 89.49 | 94.04 | 93.16 | 93.08 |
| 15 | 27.91 | 24.72 | 21.42 | 23.92 | 25.12 |
